# Contribution of *S. xylosus* and *L. sakei* ssp. *carnosus* Fermentation to the Aroma of Lupin Protein Isolates

**DOI:** 10.3390/foods10061257

**Published:** 2021-06-01

**Authors:** Katharina Schlegel, Eva Ortner, Andrea Buettner, Ute Schweiggert-Weisz

**Affiliations:** 1 Department of Chemistry and Pharmacy, Friedrich-Alexander-Universität Erlangen-Nürnberg, 91054 Erlangen, Germany; katharina.schlegel@ivv.fraunhofer.de (K.S.); andrea.buettner@ivv.fraunhofer.de (A.B.); 2 Fraunhofer Institute for Process Engineering and Packaging (IVV), 85354 Freising, Germany; eva.ortner@ivv.fraunhofer.de; 3 Institute of Nutritional and Food Sciences, University of Bonn, 53115 Bonn, Germany

**Keywords:** lupin protein, fermentation, *Staphylococcus xylosus*, *Lactobacillus sakei* ssp. *carnosus*, gas chromatography-olfactometry, aroma, stable isotope dilution analysis, odour activity value

## Abstract

Aroma-active compounds of lupin protein isolate and lupin protein isolate fermented with *Staphylococcus xylosus* and *Lactobacillus sakei* ssp. *carnosus* were investigated. The changes in aroma-active compounds were determined by application of aroma extract dilution analysis in combination with gas chromatography-mass spectrometry/olfactometry for identification, and by stable isotope dilution assays for quantification. A total of 30 aroma-active compounds for non-fermented and fermented samples were identified. The aroma profile of LPI fermented with *Lactobacillus sakei* ssp. *carnosus* was characterized as roasty and popcorn-like. *Staphylococcus xylosus* generated cheesy impressions, being in line with the fact that the main aroma compounds acetic acid, butanoic acid, and 2/3-methylbutanoic acid could be identified. Quantification of butanoic acid further confirmed these findings with the highest concentration of 140 mg/kg for LPI fermented with *Staphylococcus xylosus*. Our study provides insights into how fermentation utilizing different fermentative microbial strains, namely *Staphylococcus xylosus* and *Lactobacillus sakei* ssp. *carnosus* alters the aroma profile of lupin protein isolates. This demonstrates the potential of shaping fermented protein-based foods via targeted microbiological refinement.

## 1. Introduction

In recent years, an increasing trend of plant-based food products can be observed in the global market. More and more people are avoiding animal products in their diet for ethical, ecological, health and animal welfare reasons. This also increases the demand for plant-based raw materials, especially proteins, in order to continue to supply the body with sufficient amounts of the essential nutrient. Legumes, such as lupins, are promising alternatives to animal protein due to their high protein content of 39% up to 55% [1] and a balanced amino acid supply [2]. Lupin proteins are already used as ingredients in various food applications, such as mayonnaise, ice cream, bakery products and pastries, milk replacements, and pasta products [3].

The use of legumes in some food is still limited due to their functional properties, such as low solubility in the acidic range. In addition, legume proteins often have a characteristic overall aroma, which is described with green, grassy and beany aroma impressions [4,5,6,7]. In particular, these distinct sensory characteristics make it difficult to use legume proteins in food and to easily substitute animal proteins with plant proteins. This problem is often overcome by adding flavouring agents to plant-based products in order to mask the typical aroma notes. The addition of many other agents—including flavourings—often leads to criticism of plant-based products. However, if it were possible to reduce the characteristic aroma notes or to create desired aroma notes by means of suitable technologies, the addition of flavourings could be reduced or even dispensed with.

Fermentation is a promising approach for modifying the aroma impressions of legumes considerably. For example, lactic acid fermentation has already been shown to decrease the green smelling n-hexanal content of pea protein extracts [8]. Other studies have shown that pea-like, green, earthy and beany aroma impressions in lupin, pea and soy protein isolate were reduced or masked by fermentation [9,10,11].

In a previous study, lupin protein isolates were fermented with *Staphylococcus xylosus* and *Lactobacillus sakei* ssp. *carnosus*, respectively, to investigate the effect of fermentation on the functional and sensory properties of lupin protein isolates [11]. Schlegel, Leidigkeit, Eisner and Schweiggert-Weisz [11] observed a remarkable change in the sensory properties of the lupin proteins fermented with *Staphylococcus xylosus* and *Lactobacillus sakei* ssp. *carnosus*. In particular, a sweaty/cheesy and a roasty/popcorn-like impression clearly emerged. Since the changes in the sensory properties of the other microorganisms used in the study were less pronounced, it was obvious to take a closer look at the aroma profile of the lupin protein isolate fermented with *Staphylococcus xylosus* and *Lactobacillus sakei* ssp. *carnosus*. Commonly, both microorganisms are commonly used as starter cultures in the fermentation of meat products [12,13,14,15,16,17,18], but their use in the fermentation of lupin proteins is, to the best of our knowledge, reported here for the first time.

The aim of this study was to investigate the changes in the main aroma compounds of lupin protein isolates after their fermentation with *Staphylococcus xylosus* and *Lactobacillus sakei* ssp. *carnosus*. Therefore, an aroma extract dilution analysis in combination with gas chromatography-mass spectrometry/olfactometry was carried out for the identification of the flavor compounds. Stable isotope dilution assays were used for their quantification.

## 2. Materials and Methods

### 2.1. Preparation of Lupin Protein Isolates and Fermented Lupin Protein Isolates

Lupin protein isolate (LPI) preparation, and fermentation of the isolate with *Staphylococcus xylosus* (*S. xylosus*, DSM 20266) and *Lactobacillus sakei* ssp. *carnosus* (*L. sakei* ssp. *carnosus,* DSM 15851) for 24 h were prepared as previously described by Schlegel, Leidigkeit, Eisner and Schweiggert-Weisz [11]. The protein content of LPI was 89.6%, and fermented LPI with *S. xylosus* and *L. sakei* ssp. *carnosus* contained of 80.1% and 80.6%, respectively [11].

### 2.2. Isolation of the Volatiles

A total 20 g (±0.3 g) of each sample were extracted using dichloromethane (DCM, 100 mL) by stirring vigorously for 30 min at room temperature in a closed vessel. After filtration, volatiles were separated from the non-volatiles using the solvent-assisted flavor evaporation (SAFE) technique according to Engel, et al. [19]. For a guaranteed careful and fast isolation of the volatile compounds, the SAFE distillation was performed using a high vacuum pump with a pressure of about 10^−4^ mbar, with a water bath temperature of 50 °C and an apparatus temperature of 55 °C. The obtained distillate was dried over anhydrous sodium sulfate, filtered and concentrated to ~3 mL at 50 °C by means of Vigreux distillation (50 cm × 1 cm i.d.), and finally to a total volume of ~100 µL by microdistillation according to Bemelmans [20].

### 2.3. Gas Chromatography-Olfactometry (GC-O)

The sample distillates were analyzed by GC-O using a Trace Ultra gas chromatograph (Thermo Fisher Scientific, Waltham, MA, USA) using a DB-FFAP (30 m × 0.32 mm, film thickness 0.25 μm, J & W Scientific, Agilent Technologies Germany GmbH & Co. KG, Waldbronn, Germany) capillary column. Aliquots (2 µL) of the distillates were manually injected by the cold on-column technique at 40 °C. After 2 min, the temperature was raised at 8 °C/min to 240 °C and held for 5 min. The flow rate of the helium carrier gas was 2 mL/min. At the end of the capillary column, the effluent was split in a 1:1 ratio into an odor detection port (ODP) and a flame ionization detector (FID). The flame ionization detector and the ODP were held at 300 °C and 250 °C, respectively. Linear retention indices (RIs) of the aroma-active compounds were calculated using a homologous series of n-alkanes (C6–C26 for DB-FFAP and C6–C18 for DB-5, respectively) as described by Van Den Dool and Kratz [21]. To avoid a potential lack of recognition of odorants, the aroma-active areas of the original distillate were evaluated by three trained panelists.

### 2.4. Aroma Extract Dilution Analysis (AEDA)

To enable a comparison between the fermented and non-fermented isolates, the same amounts were extracted, subjected to SAFE distillation, concentrated to the same final volume, and, finally, the same volume was used for gas chromatography–olfactometry (GC-O). The flavor dilution (FD) factor of each aroma compound obtained from the distillates of LPI and fermented LPI were determined using AEDA by diluting the distillate stepwise with DCM (1 + 2; *v*/*v*). GC-O analyses were performed for the undiluted distillates (FD = 1), and all subsequent dilutions. The respective FD factor assigned correlated to the highest dilution in which the compound that was still perceivable at the ODP.

### 2.5. Gas Chromatography-Mass Spectrometry/Olfactometry (GC-MS/O)

The samples were analyzed by GC-MS/O with a Trace Ultra gas chromatograph (Thermo Fisher Scientific) and a Trace DSQ mass spectrometer (Thermo Electron Corporation, Waltham, TX, USA) using a DB-FFAP (30 m × 0.32 mm, film thickness 0.25 μm, J & W Scientific, Agilent Technologies Germany GmbH & Co. KG) capillary column. Aliquots of 2 µL were injected via the cold on-column technique at 40 °C using a multipurpose autosampler MPS 2 (Gerstel GmbH & Co. KG, Mühlheim an der Ruhr, Germany). After 2 min, the temperature was raised at 8 °C/min to 240 °C, and held for 5 min. The flow rate of the helium carrier gas was 2 mL/min. At the end of the capillary column, the effluent was split into an ODP and a mass spectrometer using two deactivated fused silica capillaries (0.5 m × 0.2 mm; A-Z Analytik-Zubehör GmbH, Langen, Germany). Mass spectra in positive electron impact mode were generated at 70 eV ionization energy with a *m*/*z* range of 35–350.

### 2.6. Two-Dimensional Gas Chromatography-Mass Spectrometry/Olfactometry (2D-GC-MS/O)

The 2D-GC-MS/O system consisted of two gas chromatography systems (Agilent Technologies Germany GmbH & Co. KG) housing capillary columns with different polarities (DB-FFAP and DB-5, both 30 m × 0.32 mm, film thickness 0.25 μm, J &W Scientific, Agilent Technologies Germany GmbH & Co. KG) connected via a CTS 1 cryotrap system (Gerstel GmbH & Co. KG). A total 2 µL were injected automatically using the cold on-column technique at 40 °C by a Multipurpose Sampler MPS 2XL (Gerstel GmbH & Co. KG). At the end of the capillary column of the first GC system the effluent was split 1:1 by volume and led to an ODP (290 °C) and FID (240 °C). After 2 min, the temperature was raised at 8 °C/min to 230 °C, and held at the final temperature for 5 min in the first GC oven. The helium carrier gas flow was 8 mL/min. Afterwards, the aroma-active areas of interest were transferred to the cryogenic trap (−100 °C). After thermodesorption in the second GC system at 250 °C, the volatiles were transferred to the second column at a starting temperature of 40 °C. The temperature was raised at 8 °C/min to a final temperature of 250 °C and held for 1 min. At the end of the capillary of the second GC system, the effluent was split and passed in equal parts to both an ODP and a MS. Mass spectra were recorded in positive electron impact ionization mode at 70 eV ionization energy. The *m*/*z* range was 35–399.

### 2.7. Quantification of Aroma-Active Compounds

The quantification of the selected aroma compounds acetic acid, butanoic acid, 2-acetyl-1-pyrroline, 4-hydroxy-2,5-dimethyl-3(*2H*)-furanone and 3-(methylthio)propanal in LPI and fermented LPI was carried out using stable isotope dilution assays. Different amounts of sample material (1–5 g; depending on the concentrations of the respective odorants determined in preliminary experiments) were used for quantitation of the aroma-active compounds. The isotopically labeled internal standards were dissolved and stored in dichloromethane.

A suspension of the respective sample material, 100 mL DCM and the labeled internal standards (amounts depending on the concentrations of the analytes) were stirred (300 rpm) for 30 min at room temperature in a closed vessel. Further workup was performed as described above for the isolation of the volatiles. The quantitation experiments were performed in duplicates. The quantitation was performed via gas chromatography−mass spectrometry (GC-MS) or two-dimensional high-resolution gas chromatography−mass spectrometry (2D-GC-MS) as described above.

Determination of response factors: for each odorant, a response factor was calculated by analyzing binary mixtures of defined amounts of the unlabeled analyte and the labeled standard in five different mass ratios (5:1, 3:1, 1:1, 1:3, 1:5) under the same chromatography conditions used for the samples.

### 2.8. Orthonasal Odor Thresholds (OT)

For the calculation of odor activity values (OAVs), orthonasal odor thresholds (OTs) of selected aroma compounds were taken from Czerny, et al. [22].

## 3. Results

### 3.1. Identification of Aroma-Active Volatile Organic Compounds (VOC)

First, the volatiles were extracted from the protein isolates by DCM followed by high vacuum distillation using the SAFE technique. The distillates obtained of each kind of protein isolate exhibited the typical characteristic overall aroma, when a drop of the distillate was put on a strip of filter paper, proving the successful extraction of all key aroma compounds. Next, the distillates were subjected to AEDA as a screening method for differentiation between aroma-active VOC and the bulk of odorless VOC. Application of AEDA by GC-O revealed in total 30 aroma-active regions for all samples in an FD factor range between 1 and ≥6561 (Table 1). The GC-O analyzes of the non-fermented lupin protein isolate (LPI) distillate resulted in the detection of 28 aroma-active regions in the undiluted distillate (FD 1) performing the GC-O analyses on a DB-FFAP capillary column. Six of these compounds (no. 23, 25, 26, 27, 29, 30) were still perceivable in the highest FD factor of 729. However, it is important to note, that the most intense aroma-active regions did not correlate with the highest FID peak signals. Trace substances may belong to the potent aroma contributors whereas the quantitative dominance of a volatile substance may not be directly correlated with an overall aroma impact.

Analysis of the distillates obtained from the LPI fermented with *S. xylosus* and *L. sakei* ssp. *carnosus* resulted in the detection of 30 aroma-active regions in each of the respective undiluted distillates (FD 1). Three aroma-active regions (no. 15, 24, 28) in LPI fermented with *S. xylosus* were detected within the highest FD factor of ≥6561. For LPI fermented with *L. sakei* ssp. *carnosus*, five regions (no. 16 a/b, 22, 26, 29, 30) were perceived within the highest FD factor of 729. For unequivocal identification of the most potent aroma-active compounds, the respective odor quality and intensity perceived at the odor detection port, the retention indices on two capillary columns of different polarities, and mass spectra in EI mode recorded by GC-MS/O and 2D-GC-MS/O (if trace odorants coeluted with other volatiles present in higher amounts) were compared with the data of reference compounds.

As a result, in all samples fatty smelling aroma-active VOC were identified as the aldehydes (*E*)-non-2-enal (no. 10), (*Z*)-non-2-enal (no. 12), (*E*,*Z*)-nona-2,6-dienal (no. 14) and (*E*,*Z*)-nona-2,4-dienal (no. 17), whereas a metallic note was elicited by *trans*-4,5-epoxy-(*E*)-dec-2-enal (no. 15). The grassy, green and soapy smelling regions derived from hexanal (no. 1) and nonanal (no. 5). In addition, coconut-like and peach-like aroma-active regions derived from lactones such as *γ*-nonalactone (no. 20), *δ*-nonalactone (no. 23), *γ*–decalactone (no. 24), *δ*–decalactone (no. 26) and *γ*–dodecalactone (no. 28). Pea-like, green bell pepper-like aroma-active regions belonged to the class of pyrazines, such as 3-isopropyl-2-methoxypyrazine (no. 7), 3-sec-butyl-2-methoxypyrazine (no. 9) and 3-isobutyl-2-methoxypyrazine (no. 11). Cheesy and vinegar-like aroma impressions were identified as methylpropanoic acid (no. 13), butanoic acid (no. 15), 2-/3-methylbutanoic acid (no. 16 a/b), and acetic acid (no. 6). Apart from that, mushroom-like and geranium-like impressions were traced back to 1-octen-3-one (no. 2) and (*Z*)-1,5-octadien-3-one (no. 4). Moreover, 2-acetyl-1-pyrroline (no. 3; roasty/popcorn-like), 3-(methylthio)propanal (no. 8; cooked potato-like), 3-hydroxy-2-methyl-pyran-4-one (no. 18; caramel-like), octanoic acid (no. 21; musty/coriander-like/fatty), and 4-hydroxy-2,5-dimethyl-3(*2H*)-furanone (no. 22; caramel-like) were identified. In addition, nonanoic acid (no. 25; soapy), 3-hydroxy-4,5-dimethylfuran-2(*5H*)-one (no. 27; savory-like), 4-hydroxy-3-methoxy-benzaldehyde (no. 30; vanilla-like), and phenylacetic acid (no. 29; bee wax-like/honey-like) were successfully identified in the lupin protein isolates.

### 3.2. Quantification of Aroma-Active Compounds by Stable Isotope Dilution Assays (SIDA)

Next, five selected odorants either based on the main aroma impressions previously described in sensory analyses using comparative aroma profile analyses or differing in their FD factor during AEDA (Table 1) were quantified in non-fermented and fermented LPI by means of SIDAs using the respective stable isotopically labeled internal standards (Table 2).

Quantification revealed the highest concentration for acetic acid in all samples with the highest amount of 2500.0 mg/kg for *S. xylosus* (Table 3). For LPI fermented with *L. sakei* ssp. *carnosus* and non-fermented LPI concentrations of 83.0 mg/kg and 13.0 mg/kg were determined, respectively. Butanoic acid was quantified in the samples fermented with *S. xylosus* and *L. sakei* ssp. *carnosus* with amounts of 140.0 mg/kg and 15.0 mg/kg, respectively, and with a concentration of 570.0 µg/kg for non-fermented LPI. 2-Acetyl-1-pyrroline was present in concentrations of 15.0 µg/kg (LPI), 14.0 µg/kg (*S. xylosus*) and 14.0 µg/kg (*L. sakei* ssp. *carnosus*) and 3-(methylthio)propanal with 2.8 µg/kg (*S. xylosus*), 2.7 µg/kg (LPI), and 2.4 µg/kg (*L. sakei* ssp. *carnosus*). The 4-Hydroxy-2,5-dimethyl-3(*2H*)-furanone was quantified in the fermented samples *S. xylosus* and *L. sakei* ssp. *carnosus* with amounts of 45.0 µg/kg and 50.0 µg/kg, respectively, but were found to be below the limit of quantification (LOQ) for non-fermented LPI.

### 3.3. Odor Activity Values (OAV)

OAVs are defined as the ratio of the concentration of an aroma substance in a sample to its odor threshold and are indicative of the relative importance of an individual aroma substance to the overall aroma. OAVs were calculated for the five quantified aroma compounds (Table 3) using odor recognition thresholds in water taken from Czerny et al. [22] and are shown in Table 4.

The highest OAV was calculated for 2-acetyl-1-pyrroline for all samples with values of 124 for LPI and 113 for *S. xylosus* and *L. sakei* ssp. *carnosus* fermented samples. Further, butanoic acid was found with an OAV of 18 for the sample fermented with *S. xylosus*, and was, therefore, potentially of higher sensory impact than the samples fermented with *L. sakei* ssp. *carnosus* (OAV 2) and LPI (OAV < 1). In addition, for acetic acid, an OAV > 1 was only achieved for *S. xylosus* (OAV 14), whereas an OAV of 2 was calculated for the remaining samples. The 4-Hydroxy-2,5-dimethyl-3(*2H*)-furanone did not exceed an OAV > 1 in any of the investigated samples.

## 4. Discussion

### 4.1. Assignment of Individual Aroma-Active Compounds to the Descriptive Aroma Impression of the Fermented Lupin Protein Isolates

The comparison of the AEDA results obtained in this study with the results from the comparative aroma profile analyses of non-fermented LPI and LPI fermented with *S. xylosus* and *L. sakei* ssp. *carnosus* as previously reported by Schlegel, Leidigkeit, Eisner and Schweiggert-Weisz [11] are in very good agreement. In our previous study, non-fermented LPI was primarily characterized using descriptive analyzes [11] by the retronasal aroma attributes pea-like/green bell pepper-like and oatmeal-like/fatty, followed by earthy/moldy/beetroot-like, cooked potato-like and roasty/popcorn-like aroma impressions. LPI fermented with *S. xylosus* exhibited an intense cheesy aroma impression followed by a moderate intensity of oatmeal-like/fatty impressions. LPI fermented with *L. sakei* ssp. *carnosus* was described with popcorn-like/roasty as main aroma characteristic, followed by oatmeal-like/fatty with a moderate intensity.

The aroma attributes during aroma profiling pea-like/green bell pepper-like for LPI could be related to the presence of 3-isopropyl-2-methoxypyrazine (no. 7; pea-like/green bell pepper-like), 3-sec-butyl-2-methoxypyrazine (no. 9; pea-like/green bell pepper-like), and 3-isobutyl-2-methoxypyrazine (no. 11; pea-like/green bell pepper-like). The identified components (*E*)-non-2-enal (no. 10; fatty/cardboard-like), (*Z*)-non-2-enal (no. 12; cardboard-like/fatty) and (*E*,*Z*)-nona-2,4-dienal (no. 17; fatty/cucumber-like/cardboard-like) in LPI are likely to be responsible for the fatty aroma impression obtained during aroma profiling. Similar impressions and components were also detected in the samples fermented with *S. xylosus* and *L. sakei* ssp. *carnosus*. The aroma impression cooked potato-like and roasty/popcorn-like can be attributed to the presence of the components 3-(methylthio)propanal (no. 8; cooked potato-like) and 2-acetyl-1-pyrroline (no. 3; roasty/popcorn-like), respectively.

For all the mentioned aroma-active VOC for LPI, an FD factor of 9 was determined. Although the FD factor of the individual components was not particularly high, roasty/popcorn-like and cooked potato-like impressions seem to have a high relevance for the overall aroma profile of LPI. Possibly, the presence of odorants with similar aroma impressions added up to yield the intensity of the overall sensory impression [23,24]. Additive and synergistic effects as well as suppressive effects of individual odorants are very common in aroma substance mixtures, but are not captured in the course of GC-O analyses, and can only be elaborated by additional sensory tests. The intense aroma impression cheesy in the sample fermented with *S. xylosus* was thereby likely due to the combined presence of methylpropanoic acid (no. 13; cheesy/sweaty), butanoic acid (no. 15; cheesy) and 2-/3-methylbutanoic acid (no. 16 a/b; sweaty/cheesy). The high FD factor of >6561 for butanoic acid and of 729 for 2-/3-methylbutanoic acid provide further evidence for a high relevance of the two components for the overall aroma profile of fermented LPI with *S. xylosus*, corresponding with the pronounced intensity of the sweaty-cheesy aroma impression in the sensory evaluation. The main aroma impression roasty/popcorn-like of LPI fermented with *L. sakei* ssp. *carnosus* in sensory analysis can likewise come from 2-acetyl-1-pyrroline (no. 3; roasty/popcorn-like).

In summary, the sensory data strongly corresponded with the odorants identified here, as well as the AEDA results in so far as the fermented samples showed stronger sensory impressions of sweaty/cheesy (*S. xylosus*) and roasty/popcorn-like (*L. sakei* ssp. *carnosus*), respectively, corresponding with the observation that the AEDA showed higher FD factors for these odorants in the fermented samples compared to non-fermented LPI.

### 4.2. Comparison of FD Factors of Fermented and Non-Fermented Lupin Protein Isolates and Quantification of Individual Aroma Compounds

When comparing the identified components and respective FD factors of LPI and LPI fermented with *S. xylosus* and *L. sakei* ssp. *carnosus*, it can be further observed that the FD factors of the aroma-active acids were higher in the fermented samples than in non-fermented LPI. Acetic acid (no. 6) with an FD 9 for LPI was determined with a higher FD in the sample fermented with *S. xylosus* (FD 729) and with *L. sakei* ssp. *carnosus* (FD 27), which was supported by the quantitative data. LPI had a concentration of 13.0 mg/kg, compared to 83.0 mg/kg for fermented LPI with *L. sakei* ssp. *carnosus* and a concentration of 2500.0 mg/kg acetic acid for the one fermented with *S. xylosus*. Correspondingly, with an OAV of 14 in samples fermented with *S. xylosus*, this compound was more pronounced in the same sample than in samples fermented with *L. sakei* ssp. *carnosus* and non-fermented LPI (both OAV < 1). Moreover, 2-and 3-methylbutanoic acid (no. 16 a/b) were characterized with an FD factor of 729 in the fermented LPI, being higher than in non-fermented LPI with an FD of 9. Butanoic acid (no. 15) had an FD factor of ≥6561 in LPI fermented with *S. xylosus*, being likewise considerably higher than in the sample fermented with *L. sakei* ssp. *carnosus* (FD 243). Butanoic acid and methylpropanoic acid (no. 13) were not detectable in the undiluted distillate of non-fermented LPI using GC-O. Quantitative analyses of butanoic acid confirmed the higher FD factor for LPI fermented with *S. xylosus* compared to *L. sakei* ssp. *carnosus* with concentrations of 140.0 mg/kg to 15.0 mg/kg, respectively, whereas for LPI, a concentration of 570.0 µg/kg was determined. In addition, results of the OAV calculation showed values of 18 for samples fermented with *S. xylosus*, and 2 for *L. sakei* ssp. *carnosus*. In this case, the OAV for LPI was <1. The higher concentration of butanoic acid in the *S. xylosus* fermented sample was also consistent with sensory profiling, where the aroma impression cheesy was the main impression of the *S. xylosus* fermented sample. Nevertheless, LPI fermented with *L. sakei* ssp. *carnosus* also contained butanoic acid and 2/3-methylbutanoic acid (same FD as *S. xylosus*). However, these components do not appear to be of major relevance to the overall aroma profile of the *L. sakei* ssp. *carnosus* fermented sample. In addition, higher FD levels of 4-hydroxy-2,5-dimethyl-3(*2H*)-furanone (no. 22) were determined in LPI fermented with *S. xylosus* (FD 81) and *L. sakei* ssp. *carnosus* (FD 729), than in LPI (FD 3). In this respect, the results of the quantitative analysis were in good agreement and showed concentrations of 50.0 µg/kg for the *L. sakei* ssp. *carnosus* fermented sample and 45.0 µg/kg for the *S. xylosus* fermented sample, whereas levels < LOQ (19.7 µg/kg) were detected in non-fermented LPI. Accordingly, the calculated OAV was for all samples <1 and therefore most likely not of relevance for the overall aroma, although 4-hydroxy-2,5-dimethyl-3(*2H*)-furanone was detected during AEDA. This is due to the fact, that in AEDA the influence of the matrix on the aroma release is not effective.

While the results of the sensory analysis for LPI fermented with *L. sakei* ssp. *carnosus* showed roasty/popcorn-like as the main aroma impression, only differences in one FD range for 2-acetyl-1-pyrroline (no. 3) was detected between LPI fermented with *L. sakei* ssp. *carnosus* (FD 9), fermented with *S. xylosus* (FD 3) and non-fermented LPI (FD 3). This was also confirmed by the quantitative analysis of 2-acetyl-1-pyrroline by SIDA, where LPI fermented with *L. sakei* ssp. *carnosus* and *S. xylosus* showed a concentration of 14.0 µg/kg each. The highest concentration of 2-acetyl-1-pyrroline was 15.0 µg/kg for LPI. The odor threshold for 2-acetyl-1-pyrroline is 0.12 µg/L [22], resulting in relatively high OAV values despite their low concentrations in the samples. Accordingly, the obtained OAV indicates, with values of 124 for LPI and 113 for the samples fermented with *S. xylosus* and *L. sakei* ssp. *carnosus*, an overall contribution in all samples. Thereby, it can be assumed that the roasty/popcorn-like impression was more prominent in the fermented sample with *L. sakei* ssp. *carnosus* may be due to additive or suppressive effects of other matrix ingredients. Likewise, the aroma profile of LPI was described with a moderate intensity of a cooked potato-like note but only weak intensities of this aroma impression for fermented samples with *S. xylosus* and *L. sakei* ssp. *carnosus.* However, comparison of the FD factors and concentrations of 3-(methylthio)propanal (no. 8) revealed, only minor differences between all samples. The odor threshold for 3-(methylthio)propanal is 1.4 µg/L [22], and the resulting OAV of 2 indicates overall potential contribution, even if not pronounced, of this characteristic note to the aroma profiles of all samples. It can be assumed that the higher cooked potato-like impression in non-fermented LPI is also due to additive or suppressive effects of the matrix, as well as due to the lower acid content in non-fermented LPI to fermented LPI, resulting in improved prominence of such notes. 

### 4.3. Potential Formation of Aroma-Active Compounds

*S. xylosus* and *L. sakei* ssp. *carnosus* are widely used as starter cultures in meat and sausage fermentation. Their metabolic pathways and products as well as the formation of aroma-active VOC have already been described in numerous studies. Acetic acid is a typical aroma compound of dry fermented sausages [12,13,15,17,25,26,27]. The formation may result from side reactions of homofermentative carbohydrate metabolism (e.g., alternative degradation of pyruvate) that occur in lactic acid bacteria as well as in staphylococci [12,26]. Another way of acetic acid production is by fatty acid oxidation and by alanine catabolism [28]. Butanoic acid is often identified in meat fermentation with *S. xylosus* und *L. sakei* ssp. *carnosus* and sausage products [12,13,25]. It is known that butanoic acid is formed from pyruvate, which is produced during the metabolism of sugars. 2- and 3-methylbutanoic acid and 2-methylpropanoic acid belong to the commonly occurring important aroma compounds of dry fermented sausage with *S. xylosus* and *L. sakei* ssp. *carnosus* [13,16,17,18,25,29,30]. Branched-chain amino acids (BCAAs), such as leucine, isoleucine, and valine, are thereby the main source of 2- and 3-methylbutanoic acid and 2-methylpropanoic acid [13,14,16,31,32]. Leucine is present in LPI from *Lupinus angustifolius* L. cultivar Boregine with a content of about 7% and isoleucine and valine with contents of about 4% and 3%, respectively [2]. In the present study, the degradation of amino acids was not investigated. Therefore, no conclusion on the course of the biosynthetic pathways and their modulation by metabolic processes can be drawn. Apart from that, *δ*- and *γ*-nonalactone, -decalactone and dodecalactone are important aroma compounds in some fermented fruit, dairy, and meat products [33]. They can be formed from free fatty acids during fermentation [33], or they can be generated thermally induced from *δ*- and *γ*-hydroxy acids or triglycerides [34]. Possibly, the higher FD factors of the aroma components *γ*-nonalactone, *γ*-decalactone, and *γ*-dodecalactone in fermented LPI with *S. xylosus* is due to an intensified formation of free fatty acids during fermentation. However, such postulated formation pathways would require more detailed investigation of the formation/degradation of free fatty acids. Finally, 2-acetyl-l-pyrroline and 4-hydroxy-2,5-dimethyl-3(*2H*)-furanone have been described as thermally induced volatiles from proline [35,36] and sugar [37], respectively, [35] and were reported in several foods such as dairy products, popcorn and bread [35,36,37]. Their occurrence is, accordingly, likewise plausible, and completes our overall understanding of the characterizing odorants that form the aroma profiles of the fermented plant proteins.

## 5. Conclusions

Fermentation with *S. xylosus* and *L. sakei* ssp. *carnosus* affects the aroma profile of lupin protein isolate. Identification of aroma-active VOC by GC-O in fermented lupin protein isolate showed similarities to aroma compounds formed during fermentation of meat and sausage products (acetic acid, butanoic acid, 2-/3-methylbutanoic acid). Nevertheless, the aroma profile was characterized as cheesy after fermentation with *S. xylosus* and as roasty, popcorn-like with *L. sakei* ssp. *carnosus*. Lupin protein isolate fermented with *S. xylosus* could therefore be a promising approach for the production of plant-based cheese alternatives without the addition of flavorings. *L. sakei* ssp. *carnosus* could be suitable for its use in, e.g., meat alternatives or baked goods due to its roasty aroma profile.

This study shows that fermentation could be a promising approach for the development of protein ingredients with tailor-made sensory properties. As the effect of fermentation strongly depends on the starter culture and the raw material used, it is worth investigating even more microorganisms and fermenting even more raw materials. This could expand the range of plant proteins and thus serve the ever-growing market for protein ingredients with new fermented products. These ingredients can then be used for the development of alternatives to animal products without the addition of a long ingredients list.

## Figures and Tables

**Table 1:** Aroma-active volatile organic compounds and their corresponding olfactometric and chromatographic parameters identified in aroma distillates obtained from lupin protein isolates (LPI) and with *S. xylosus* (DSM 20266) and *L. sakei* ssp. *carnosus* (DSM 15831) fermented LPI.

No. ^a^	Aroma-Active Compound	Aroma Quality ^b^	RI Value ^c^ on		FD Factor ^d^		
			DB-FFAP	DB-5	LPI	*S. xylosus*	*L. sakei* ssp. *carnosus*
1	hexanal ^f^	grassy, green	1050	800	1	1	1
2	1-octen-3-one ^e^	mushroom-like	1287	979	9	3	9
3	2-acetyl-1-pyrroline ^f^	roasty, popcorn-like	1320	932	3	3	9
4	(*Z*)-1,5-octadien-3-one ^f^	geranium-like	1252	982	1	1	1
5	nonanal ^e^	soapy	1318	1104	1	1	1
6	acetic acid ^e^	vinegar-like	1403	619	9	729	27
7	3-isopropyl-2-methoxypyrazine ^e^	pea-like, green bell pepper-like	634	1084	9	3	3
8	3-(methylthio)propanal ^f^	cooked potato-like	1398	905	9	3	3
9	3-sec-butyl-2-methoxypyrazine ^e^	pea-like, green bell pepper-like	1400	1179	3	3	3
10	(*E*)-non-2-enal ^e^	fatty, cardboard-like	1500	1188	9	1	1
11	3-isobutyl-2-methoxypyrazine ^f^	pea-like, green bell pepper-like	1500	1182	9	1	3
12	(*Z*)-non-2-enal ^e^	cardboard-like, fatty	1495	1127	9	3	3
13	methylpropanoic acid ^f^	cheesy, sweaty	1509	1937	<1	3	3
14	(*E,Z*)-nona-2,6-dienal ^e^	cucumber-like	1500	1123	9	3	3
15	butanoic acid ^e^	cheesy	1600	804	<1	≥6561	243
16 a/16 b	2-methylbutanoic acid/3-methylbutanoic acid ^e^	sweaty, cheesy	1643	861	9	729	729
17	(*E,Z*)-nona-2,4-dienal ^e^	fatty, cucumber-like, cardboard-like	1650	1189	9	81	3
18	3-hydroxy-2-methyl-pyran-4-one ^e^	caramel-like	1947	1130	3	81	1
19	*trans*-4,5-epoxy-(*E*)-dec-2-enal ^f^	metallic	1963	1375	9	729	9
20	*γ*-nonalactone ^e^	coconut-like	2012	1360	3	243	3
21	octanoic acid ^e^	musty, coriander-like, fatty	2024	1179	3	9	1
22	4-hydroxy-2,5-dimethyl-3(2*H*)-furanone ^e^	caramel-like	2035	1196	3	81	729
23	δ-nonalactone ^e^	coconut-like	2052	1360	729	3	9
24	*γ*-decalactone ^f^	peach-like	2116	1472	3	≥6561	3
25	nonanoic acid ^e^	soapy	2150	1270	729	9	81
26	*δ*-decalactone ^f^	coconut-like	2166	1501	729	729	729
27	3-hydroxy-4,5-dimethylfuran-2(5*H*)-one ^f^	savory -like	2200	1102	729	243	243
28	*γ*-dodecalactone ^f^	peach-like, flowery	2334	1680	9	≥6561	9
29	phenylacetic acid ^f^	bee wax-like, honey-like	2510	1256	729	729	729
30	4-hydroxy-3-methoxy-benzaldehyde ^e^	vanilla-like	2524	1395	729	729	729

^a^ Odorants were consecutively numbered according to their retention indices on capillary DB-FFAP. ^b^ Aroma quality perceived at the odor detection port. ^c^ Retention index calculated according to Van Den Dool and Kratz [21]. ^d^ Flavor dilution factor determined by AEDA on capillary DB-FFAP. ^e^ The compound was identified by the comparison of the aroma quality, the RI on two capillary columns with different polarity, and mass spectrum obtained by 2D-GC-MS, compared to the reference compound. ^f^ The compound was identified by the comparison of the aroma quality and the RI at a DB-FAP and a DB-5 with the reference compound.

**Table 2:** Selected ions (*m*/*z*) of analytes and stable isotopically labeled internal standards as well as response factors (Rf) used in stable isotope dilution assays for quantitation of five selected odorants in non-fermented LPI and fermented LPI with *S. xylosus* (DSM 20266) and *L. sakei* ssp. *carnosus* (DSM 15831).

Aroma-Active Compound	Isotope Label	Ion (*m*/*z*)		R_f_
		Analyte	Standard	
acetic acid	^13^C_2_	60	62	0.99
butanoic acid	^13^C_2_	88	90	0.74
2-acetyl-1-pyrroline	^2^H_3–5_	111	114–116	0.89
4-hydroxy-2,5-dimethyl-3(*2H*)-furanone	^13^C_2_	128	130	1.01
3-(methylthio)propanal	^2^H_3_	48	51	1.08

**Table 3:** Concentration of five selected aroma-active compounds in non-fermented LPI and with *S. xylosus* (DSM 20266) and *L. sakei* ssp. *carnosus* (DSM 15831) fermented LPI determined using stable isotope dilution analysis (SIDA).

Aroma Compound	Concentration (µg/kg)					
	LPI		*S. xylosus*		*L. sakei* ssp. *carnosus*	
	Mean ^a^	Range ^b^	Mean ^a^	Range ^b^	Mean ^a^	Range ^b^
acetic acid	13,000.0	9100.0–15,000.0	2,500,000.0	2,100,000.0–2,900,000.0	83,000.0	77,000.0–87,000.0
butanoic acid	570.0	330.0–960.0	140,000.0	120,000.0–150,000.0	15,000.0	13,000.0–19,000.0
2-acetyl-1-pyrroline	15.0	15.0	14.0	13.0–14.0	14.0	13.0–14.0
4-hydroxy-2,5-dimethyl-3(*2H*)-furanone	<LOQ ^c^	<LOQ	45.0	44.0–46.0	50.0	33.0–77.0
3-(methylthio)propanal	2.7	2.6–2.8	2.8	2.7–3.0	2.4	2.3–2.5

^a^ Mean value calculated from *n* (*n* = 3) determinations. ^b^ Concentration range from *n* determinations. ^c^ Limit of quantification, LOQ ≤ 19.7 µg/kg.

**Table 4:** Odor activity values (OAVs) of six selected aroma compounds calculated based on odor recognition thresholds (OT).

Aroma Compound	OT (µg/L) ^a^	OAV		
		LPI	*S. xylosus*	*L. sakei* ssp. *carnosus*
acetic acid	180,000.0	<1	14	<1
butanoic acid	7700.0	<1	18	2
2-acetyl-1-pyrroline	0.1	124	113	113
4-hydroxy-2,5-dimethyl-3(*2H*)-furanone	160.0	<1	<1	<1
3-(methylthio)propanal	1.4	2	2	2

^a^ Orthonasal odor thresholds in water taken from Czerny et al. [22].
